# Macrophage Polarization and Reprogramming in Acute Inflammation: A Redox Perspective

**DOI:** 10.3390/antiox11071394

**Published:** 2022-07-19

**Authors:** Salvador Pérez, Sergio Rius-Pérez

**Affiliations:** Department of Physiology, Faculty of Pharmacy, University of Valencia, Burjasot, 46100 Valencia, Spain

**Keywords:** macrophage polarization, inflammation, oxidative stress, redox signaling

## Abstract

Macrophage polarization refers to the process by which macrophages can produce two distinct functional phenotypes: M1 or M2. The balance between both strongly affects the progression of inflammatory disorders. Here, we review how redox signals regulate macrophage polarization and reprogramming during acute inflammation. In M1, macrophages augment NADPH oxidase isoform 2 (NOX2), inducible nitric oxide synthase (iNOS), synaptotagmin-binding cytoplasmic RNA interacting protein (SYNCRIP), and tumor necrosis factor receptor-associated factor 6 increase oxygen and nitrogen reactive species, which triggers inflammatory response, phagocytosis, and cytotoxicity. In M2, macrophages down-regulate NOX2, iNOS, SYNCRIP, and/or up-regulate arginase and superoxide dismutase type 1, counteract oxidative and nitrosative stress, and favor anti-inflammatory and tissue repair responses. M1 and M2 macrophages exhibit different metabolic profiles, which are tightly regulated by redox mechanisms. Oxidative and nitrosative stress sustain the M1 phenotype by activating glycolysis and lipid biosynthesis, but by inhibiting tricarboxylic acid cycle and oxidative phosphorylation. This metabolic profile is reversed in M2 macrophages because of changes in the redox state. Therefore, new therapies based on redox mechanisms have emerged to treat acute inflammation with positive results, which highlights the relevance of redox signaling as a master regulator of macrophage reprogramming.

## 1. Introduction

Macrophages are tissue-resident or infiltrated immune cells that perform essential functions in both tissue homeostasis and inflammation [1,2]. The phenotype and functions of macrophages are heterogeneous and critically depend on the surrounding microenvironment [3]. In this review, we summarize how redox signals regulate macrophage polarization and reprogramming in the acute inflammation context. We also address the therapeutic opportunity of macrophage reprogramming in light of redox biology.

### 1.1. Macrophage Polarization

**Figure 1:**
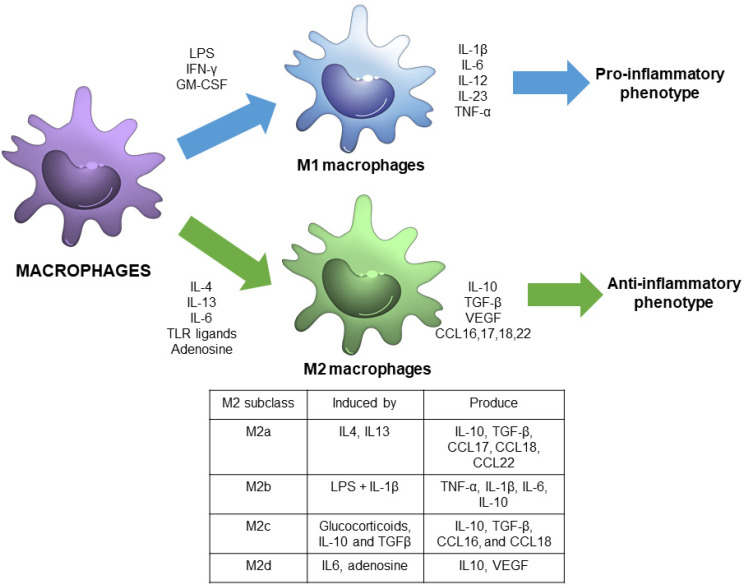
Schematic view of macrophage polarization showing the main signals and responses associated with M1 and M2 phenotypes, including M2 subdivisions.

Macrophage polarization refers to the process by which macrophages produce distinct functional phenotypes as a reaction to specific microenvironmental signals. It is considered that macrophages can polarize in two distinct subsets: M1 or M2 macrophages [4] (Figure 1).

M1 macrophages, also called classically activated macrophages, are polarized by lipopolysaccharide (LPS), either alone or in association with Th1 cytokines, such as interferon (IFN)-γ and granulocyte-macrophage colony-stimulating factor (GM-CSF). As a result, M1 macrophages produce pro-inflammatory cytokines, including interleukin-1β (IL-1β), IL-6, IL-12, IL-23, and tumor necrosis factor-α (TNF-α) through the activation of different transcription factors, such as the signal transducer and activator of transcription 1 (STAT1), nuclear factor-kappa light chain-enhancer of activated-B cells (NF-κB) and interferon regulatory factor 5 (IRF-5) [3,5]. Therefore, M1 macrophages are associated with a pro-inflammatory phenotype [6]. 

In contrast, M2 macrophages, also called alternatively activated macrophages, are polarized by Th2 cytokines, such as IL-4 and IL-13, and have an anti-inflammatory and immunoregulatory phenotype [3]. M2 macrophages produce anti-inflammatory cytokines -IL-10, transforming growth factor (TGF-β), among others, through the activation of several transcription factors, including STAT3, STAT6, IRF-4, and peroxisome proliferator-activated receptor (PPAR)-γ [5]. 

In the past decade, M2 macrophages have been further subdivided into four subgroups, M2a, M2b, M2c, and M2d, in accordance with their in vitro upstream activators and downstream gene expression patterns [7]. M2a macrophages are activated by IL-4 and IL-13, and exhibit increased expression of IL-10, TGF-β, C-C motif chemokine ligand (CCL) 17, CCL18, and CCL22. M2b are induced by Toll-like receptor (TLR) ligands and IL-1β, and express TNF-α, IL-1β, IL-6, and IL-10. M2c are activated by glucocorticoids, IL-10 and TGFβ, and exhibit increased transcription of IL-10, TGF-β, CCL16, and CCL18. M2d are induced by IL-6 and adenosine, and enhance the expression of IL10 and vascular endothelial growth factor (VEGF) [4,7].

These classifications show the complex nature of macrophages, which are plastic cells capable of modifying their gene transcription profile along a continuous spectrum, especially in pathological situations. Therefore, M1 and M2 macrophages are considered the extremes of a continuous set of heterogeneous functional phenotypes that are dynamically activated during the course of inflammatory disorders [8,9]. 

### 1.2. Role of Macrophage Polarization in Acute Inflammatory Processes

The M1/M2 macrophage balance strongly affects the progression of inflammatory processes [3]. M1 macrophages are implicated in initiating and sustaining inflammation, which is counteracted by M2 macrophages [10]. 

During inflammation, macrophages first exhibit the M1 phenotype to release TNF-α, IL-1β, IL-12, and IL-23 to neutralize the pro-inflammatory stimulus. However, if the M1 phase continues, it can cause tissue damage [3]. To avoid this, M2 macrophages secrete large amounts of IL-10 and TGF-β to suppress the inflammatory cascade, contribute to tissue repair, favor tissue remodeling, initiate vasculogenesis, and preserve tissue homeostasis. Therefore, M2 macrophages are associated with the resolution of inflammation and the initiation of tissue repair [3,11]. 

It is noteworthy that the coordinate action of various inflammatory modulators, signaling molecules and transcription factors is critical for regulating macrophage polarization, a highly dynamic process that occurs during inflammatory disease [10]. Here, we briefly summarize the dynamic changes of macrophage polarization and its impact on disease progression under some acute inflammatory conditions.

#### 1.2.1. Sepsis

Sepsis is a life-threatening condition characterized by a patient’s immune response to a severe infection that can lead to systemic inflammation and multiple organ damage. As in any type of infection, macrophages regulate the host’s immune response through their polarization to different functional phenotypes, and the dysfunction of this immune response is one of the mechanisms that underlies the onset and development of sepsis. [12]. After infection, and in the early sepsis stage, macrophages are activated through TLR [13] and differentiate into M1 by activating NF-κB, which drives the release of large amounts of pro-inflammatory cytokines, such as TNF-α, IL-1, IL-6, and IL-8 [12]. In this stage, host defense is promoted to eliminate invading pathogen or damaged tissues [14]. Then, in the late phase, macrophages polarize to the M2 phenotype by repressing NF-κB activation via the p50 NF-κB subunit to dampen the pro-inflammatory response [15]. 

It is important to note that if macrophages are excessively activated or not adequately regulated in the early sepsis phase, a cytokine storm may occur [12]. Cytokine storm is one of the main causes of the high mortality rate in sepsis because it generates an amplified and uncontrolled inflammatory response that leads to extensive tissue damage and multi-organ failure [16]. Therefore, the regulation of macrophage polarization during the devastating phase of the inflammatory response of sepsis has been considered by different authors as a key element in this pathology. The general paradigm is based on the fact that the inhibition of M1 macrophages can significantly reduce tissue damage and mortality due to the suppression of the release of inflammatory factors [17,18,19]. On the other hand, and complementing these works, it has also been seen that driving towards an M2 phenotype can also reduce inflammatory factors and improve survival rates [20,21,22].

However, for a complete understanding of the pathogenesis of sepsis, more factors must be taken into account. Recent studies have shown that both pro-inflammatory and anti-inflammatory responses occur early and simultaneously in sepsis, and, therefore, the inhibition or activation of the different phenotypes may not be effective depending on the context [23]. A change in the phenotype can also favor pathogens to develop strategies that interfere with the phagocytic function of M1 macrophages [24]. Indeed, in an interesting study, Carestia et al. demonstrated in E. coli-induced sepsis model that reprogramming monocytes towards the M1 phenotype reduced mortality by increasing iNOS expression and bacterial clearance [25]. 

Furthermore, along with these innate immune system responses, there is also a decline in the adaptive immune response, which if not resolved can trigger immunosuppression and lead to death within days [16]. Sepsis-induced immunosuppression is due to lymphopenia and loss of immune function resulting from a loss of B cells and T cells via apoptosis caused by excess cytokines [26]. Recently, it has also been observed that the presence of myeloid-derived suppressor cells (MDSCs) contributed to the inactivation of the adaptive immune system by inhibiting T cell proliferation in the early stage of sepsis [27]. 

Taken together, these works show that, although macrophage polarization is a key element in sepsis, there are numerous overlapping mechanisms that demonstrate the complexity of this pathology and its difficult approach.

#### 1.2.2. Viral Infections

After viral infection, macrophages can phagocyte viruses to present viral peptides via the major histocompatibility complex (MHC) to T-lymphocytes [6]. Then, T-lymphocytes produce IFN-γ and other inflammatory signals to induce an inflammatory response to avoid viral replication. In this viral infection stage, macrophages are polarized to M1. However, macrophages phenotype switch is activated later to suppress inflammation and to clear apoptotic cells in infected tissues [28,29]. 

It is noteworthy that macrophage polarization can be manipulated by viruses during infection to promote M2 polarization. This situation may limit virus clearance by repressing pro-inflammatory and CD4 T cell responses at infection sites [28].

#### 1.2.3. Acute Kidney Injury

Glomerular and interstitial macrophage infiltration can be detected in acute kidney injury [30]. Pro-inflammatory M1 macrophages are predominant in early-stage kidney diseases and are the first responders to kidney injury. These macrophages stimulate leukocyte infiltration and cytokine secretion, and produce cytotoxic agents, such as reactive oxygen species (ROS), which induce mitochondrial damage and apoptosis in kidney cells. Thus severe kidney damage occurs when inflammation remains unresolved by M2 macrophages [7,30]. 

M2 macrophages can secrete anti-inflammatory cytokines, resolvins, lipoxins, and matrix metalloproteinases that mediate the cleavage of chemokines and chemo-attractants, which leads to the inhibition of inflammatory cell activity [7]. In addition, M2 macrophages induce regulatory T-cells to exert their immunosuppressive effects, secrete trophic factors, promote angiogenesis, and accelerate the repair of endothelial injury in inflamed kidneys [30,31,32]. Macrophage-derived wingless-related integration site (Wnt) 7b signaling can accelerate the renewal of stem or progenitor cells, and promote epithelial responses that lead to kidney repair [33]. Therefore, the immune activity of M2 macrophages accelerates the resolution of inflammation and the repair process. However, excessive and prolonged M2 macrophages activation can promote kidney fibrosis via the secretion of TGF-β1 [34,35]. Hence, failure in the macrophages switch between a pro-inflammatory M1 and a reparative M2 phenotype may induce progressive renal inflammation and fibrosis [30].

#### 1.2.4. Acute Liver Injury

Hepatic macrophages, resident and recruited, represent 90% of all macrophages in the human body and their dysregulation is critically involved in the development of several liver diseases, such as fatty liver disease, hepatitis, fibrosis, hepatocellular carcinoma, and acute liver injury (ALI) [36]. ALI is the acute hepatic inflammation caused by endotoxin, certain drugs, or other physicochemical factors, which may trigger liver dysfunction or even acute liver failure [37]. As in the other acute diseases discussed, recent studies provide ample evidence that macrophage polarization also plays a key role in the initiation and development of ALI [38]. Experimentally, D-galactosamine, thioacetamide, LPS, and acetaminophen (APAP) have been used to produce ALI models to study macrophage plasticity in this context. 

In general, it has been seen that the STAT1 and NF-kB signaling pathways are related to the activation of M1 macrophages, while the STAT6 pathway is specific to M2 macrophages in the different models [39,40,41]. However, in APAP-induced ALI, complementary pathways to those mentioned have been observed and have helped to clarify new mechanisms in this acute pathology. Rada et al. have demonstrated that sirtuin 1 (SIRT1) protein levels are downregulated by IL1β/NFκB signaling in APAP-induced hepatoxicity, resulting in inflammation and oxidative stress [42]. Furthermore, in other studies, hyperglycemia has also been seen to aggravate ALI after treatment with APAP by promoting liver-resident macrophage proinflammatory via AMPK/PI3K/AKT-mediated oxidative stress [43]. Although these works address different pathways, both propose that the increase in M2 macrophage polarization or the inhibition of M1 polarization may be a therapeutic target to alleviate ALI.

#### 1.2.5. Acute Pancreatitis

Macrophages play a critical role in the pathogenesis of acute pancreatitis. During acute pancreatitis, acinar cells interact with pancreatic macrophages via TNF-α, IL-1β, IL-6, and monocyte chemoattractant protein (MCP)-1 secretion, and promote the recruitment of peritoneal macrophages to pancreatic tissue [44,45]. Peritoneal macrophages exhibit M1-type activation in early-stage acute pancreatitis, releasing large amounts of pro-inflammatory cytokines and chemokines, including IL-1β, IL-8, and TNF-α, which amplifies the inflammatory cascade and leads to a systemic inflammatory response and multi-organ dysfunction [46]. In addition, the release of lipase and phospholipase from the inflamed pancreas to visceral and retroperitoneal adipose tissue favors the production of specific lipid mediators, particularly non-esterified fatty acids [47], which can cause the amplification of M1-dependent response by interfering with the polarization of macrophages to the M2 phenotype [48]. 

Remarkably, the macrophage phenotypic switch between the M1 and M2 phenotypes orchestrates inflammation and repair/regeneration processes after acute pancreatitis injury. M1 macrophages dominate in the pro-inflammatory phase of acute pancreatitis, while M2 macrophages control pancreatic repair/regeneration [49]. The regenerative process of the pancreas involves transient phases of inflammation, acinar-to-ductal metaplasia (ADM) and acinar redifferentiation [50]. ADM is characterized by acinar cell transdifferentiation into duct-like progenitor cell types, which is promoted by macrophages-derived TNF-α and regulated upon activation, normal T cell expressed and presumably secreted (RANTES) [51]. In the ADM stage, it is important to note that duct-like cells express a high prostaglandin E_2_ (PGE2) level, which promotes macrophage M2 activation via IL4Rα signaling [49]. Furthermore, phosphoinositide 3-kinase (PI3K)/protein kinase B (AKT) signaling in M2 macrophages contributes to resolve inflammation, limit the extent of ADM formation, favor acinar redifferentiation, and avoid a hyperactivated regenerative process in which sustained ECM formation would contribute to pancreatic fibrogenesis [49,50].

#### 1.2.6. Acute Respiratory Distress Syndrome

In acute respiratory distress syndrome, the transcriptional profile of alveolar macrophages is extremely dynamic [52]. Normal resident alveolar macrophages exhibit the M1 phenotype in the exudative phase of acute respiratory distress syndrome [53]. These macrophages are characterized by a high expression of pro-inflammatory genes regulated by p38 and IL-6/ janus tyrosine kinase (JAK)/STAT5 signaling, including IL-1β, IL-6, MCP-1, macrophage inflammatory protein (MIP-2), and TNF-α [52,54]. These pro-inflammatory factors are released at the inflammation site and bring about the recruitment of neutrophils from circulation into lungs and the alveolar space to, thus, promote the progression of airway inflammation, antimicrobial activity, and lung injury [55].

When causative factors of acute respiratory distress syndrome disappear, macrophages shift from the M1 phenotype to the M2 phenotype, and the disease progresses to the rehabilitation stage [53]. The release of IL-10, fibronectin 1, TGF-β, and insulin-like growth factor 1 by M2 macrophages limits the levels of proinflammatory cytokines and promote lung tissue repair. Furthermore, M2 macrophages drive the clearance of apoptotic neutrophils and debris, which contribute to overcome inflammation [53,55]. 

In the fibrotic phase of acute respiratory distress syndrome, the balance between M1/M2 macrophages determines the trend and extent of lung fibrosis. M1 macrophages promote matrix degradation by producing and/or activating matrix metalloproteinases (MMPs) in the extracellular cell matrix (ECM) [53]. M1 macrophages also release antifibrotic cytokines such as chemokine C-X-C motif ligand (CXCL10) and induce the expression of MMP-13 and MMP-3 in myofibroblast, which counteracts the fibroproliferative response in late-stage acute respiratory distress syndrome [56]. In contrast, M2 macrophages markedly express the tissue inhibitors of MMPs, which impair the removal of excessive ECM and promote ECM deposition by myofibroblast [57]. 

## 2. Redox Signaling in Macrophage Polarization

**Figure 2:**
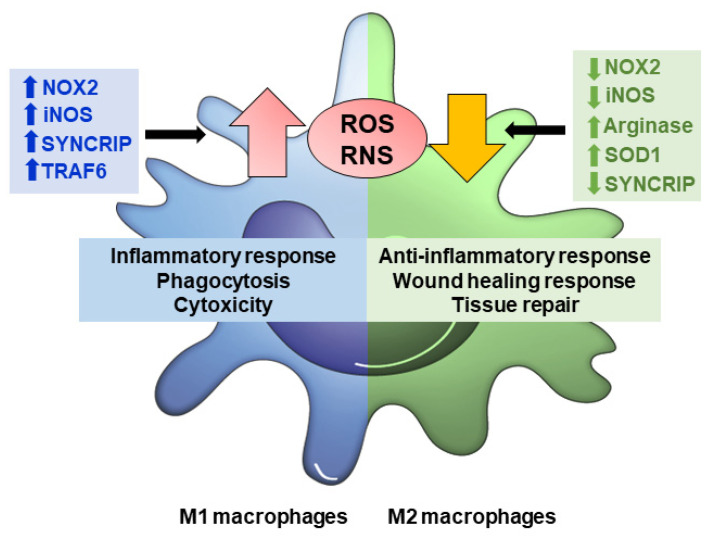
ROS/RNS production modulates macrophage polarization. In M1 macrophages, augmented levels of NOX2, iNOS, SYNCRIP (associated with NOX2 mRNA stability), and TRAF6 (involved in respiratory chain complex I stability) causes ROS/RNS production to increase, which triggers inflammatory response, phagocytosis, and cytotoxicity. M2 macrophages down-regulate NOX2, iNOS, SYNCRIP and/or up-regulate arginase and SOD1, counteract ROS and RNS, and favor anti-inflammatory response, would healing and tissue repair functions.

As previously mentioned, macrophages are known for their versatile role in physiological and pathological conditions. These processes are closely linked with the synthesis of cytokines and ROS and nitrogen species (ROS/RNS) [58]. Primarily, these oxidants species are for macrophages to remove foreign material or altered structures. However, as in other cell populations, redox-sensitive reactions can also play essential roles in differentiation, signal transduction and gene expression [59]. In fact, redox modifications and ROS/RNS production are involved in macrophage plasticity and can regulate its polarization in different subpopulations (Figure 2) [60].

### 2.1. Redox Regulation of M1 Macrophages

Stimulation with IFN-γ and LPS induces M1 macrophages activation, which are characterized by the production of large amounts of oxidants and nitrogenous species. In turn, these ROS/RNS are involved in the activation of the redox-sensitive inflammatory pathways regulated by NF-κB, p38 MAPK, and AP-1 [61,62,63]. 

In the ROS-generating systems in macrophages, one main family are NADPH oxidases (NOXs). Specifically, the presence of NOX1, NOX2, and NOX4 has been identified, with NOX2 being the best characterized during the process of M1-associated phagocytosis [64]. NOX2 consists of two multiprotein complexes: one cytosolic and one located in the membrane. The cytosolic subunit is made up of units p40phox, p47phox, and p67phox, while the cell membrane-associated part comprises NOX2 and p22phox [65]. During phagocytosis, membrane invagination first occurs to form the phagocytic vesicle. After the complete assembly of the different NOX2 subunits in the membrane of cytoplasmic phagosomes, the superoxide anion radical is synthesized, which is directly released inside the vesicle to kill the pathogen [66]. Indeed, impaired superoxide formation due to NOX2 mutations is associated with hyperinflammation and recurrent infections [64]. Furthermore, NOX2-dependent ROS generation is essential for autophagy, a key cellular process for pathogen elimination, which is activated by TLRs in M1 macrophages [67]. In fact, NOX2 deficiency has been shown to inhibit the conjugation of microtubule-associated protein 1A/1B-light chain 3 (LC3) with LC3-Associated Phagocytosis (LAP) in phagosomes and the subsequent association with lysosomal-associated membrane protein 1 (LAMP1) in macrophages exposed to *Aspergillus fumigatus* infection [68]. 

Altogether, the above-described studies highlight the essential role of NOX2 in the immune process of M1 macrophages. However, in recent years, more NOX2 activation functions other than pathogen killing have been described [69]. In particular, NOX2-derivated ROS are crucial for the monocyte to macrophage transition, and for the change in the macrophage phenotype [69]. p47phox knockout mice exhibit a reduction in the MHC class II-mediated antigen presentation (a feature of M1 macrophages) as a consequence of the alteration to the cysteine residues of cathepsins S and L [70] and, consequently, the activation of type 1 CD8^+^T-cells after *Trypanosoma cruzi* infection is compromised [71]. Therefore, taking together these results suggest that the production of ROS by NOX2 participates in key redox-based intracellular signaling mechanisms tightly associated with macrophages polarization.

Another classic system for generating ROS/RNS by macrophages is inducible nitric oxide synthase (iNOS), although macrophages may also express neuronal nitric oxide synthase (nNOS) to intensify the phagocytic function [72]. Hibbs et al. were the first to describe the antipathogenic function of macrophages through NO production by connecting the redox biology of NO to innate immunity [73]. Indeed, M1 macrophages respond to cytokines by producing large amounts of NO-oxidation products, such as nitrites and nitrates, to eliminate the pathogens responsible for infection [74]. 

Interestingly, NO provides activated macrophages cytotoxic capacity or favors the transition to the M2 phenotype, depending on NO concentrations [74]. Activated macrophages are capable of generating NO concentrations within a range that goes from nanomolar to micromolar [72], and the effects that they can exert are divided between direct and indirect depending on these concentrations. Direct effects occur at low NO concentrations (1–30 nM) and are related to nitrosylation and the subsequent activation of soluble guanylyl cyclase (sGC) [75], which cause cyclic guanosine-3′,5′-monophosphate (cGMP) levels to rise. This molecule is capable of exerting anti-inflammatory effects on macrophages [76,77] to counteract the M1 response. During direct reactions, but at concentrations between 30 and 100 nM, NO can inhibit targets such as cytochrome c oxidase (COX) or prolyl hydroxylases (PHD) to, thus, cause the blockage of the respiratory chain in pathogens and hypoxia-inducible factor (HIF)-1α stabilization under normoxic conditions, a key transcription factor that sustains the M1 metabolic signature (see Section 3.1), respectively [78,79]. When concentrations are approximately 400 nM, indirect reactions of NO occur through the formation of reactive nitrogen species, such as peroxynitrite and nitrogen dioxide, which can cause the nitration of proteins, lipids, or DNA by favoring cytotoxicity activity against pathogens [75]. Finally, when concentrations exceed 400 nM, p53 is stabilized in the nucleus due to phosphorylation, and can induce the apoptosis of M1 macrophages [80], which would lead to the resolution of inflammation and the proliferation of M2 macrophages with wound healing capacity in inflamed tissues.

Wiese et al. showed that in, apart from the induction of NOX2 and iNOS, M1 macrophages require the presence of mitochondrial ROS for their activation [81]. In this work, they generated mice with double deficiency in NOX2 and iNOS expression, and cultured the macrophages isolated from these mice under hypoxic conditions. After treating cells with different pathogens, they observed reduced activity in both control macrophages and those isolated from double KO mice. This decreased activity was the consequence of impaired mitochondrial function due to hypoxia and was further verified using respiratory chain inhibitors, which highlights the fundamental role of mitochondrial ROS in the immune function. Other works have supported the role of mitochondrial ROS in M1 macrophage activation. For example, RAW 264.7 macrophages treated with surfactin, a stimulator of mitochondrial ROS, increased TNF-α production through NF-κB activation [82]. In relation to this work, in macrophages the depletion of TNF-α receptor associated factor (TRAF) 6, involved in respiratory chain complex I stability, suppresses mitochondrial ROS production and impairs M1-associated bactericidal capacity [83]. In fact, it has been observed that deficiency in gene associated with retinoid-IFN-induced mortality 19 (GRIM-19), which belongs to the subunit of the peripheral arm of complex I, causes increased susceptibility to bacterial infection [84].

### 2.2. Redox Regulation of M2 Macrophages

Once the phagocytic function has been fulfilled by M1 macrophages, ROS production ceases to be important because tissue remodeling and wound healing functions must be established. Conversely to M1, M2 macrophages present poor proinflammatory cytokines, ROS, and NO production [85,86]. 

In the transition from M1 to M2, the decrease in ROS production is mainly mediated by the inhibition of NOX2. For example, activation of peroxisome proliferator–activated receptor gamma 1 (PPARγ1) has been shown to prevent protein kinase C alpha (PKCα) by activating NOX2 through phosphorylation in RAW 264.7 [87]. Specifically, PPARγ forms a complex with PKCα to, thus, prevent the phosphorylation of the p47phox subunit of NOX2 and, consequently, phagocytic function loss. It has also been observed how the presence of prostaglandin E2 decreases the activation of the p40phox subunit in alveolar macrophages in a mechanism that depends on PI3K/Akt pathway activation [88]. Interestingly, inhibition of the NOX2 function may not only occur through the regulation of its activity, but may also be due to mRNA stability. Specifically, the synaptogamin-binding cytoplasmic RNA interacting protein (SYNCRIP), which is capable of stabilizing NOX2 mRNA, is found in M1 macrophages. However, in M2 this protein decreases and, therefore, shortens the half-life of this mRNA [89]. At the transcriptional level, it has been reported that incubation with IL-4 results in the down-regulation of the expression of the NOX2 subunit gp91phox and in the increased expression of cathepsins S and L, which promotes the macrophage’s wound repair and tissue remodeling ability [86].

As mentioned above, the production of NO and its derivatives is a typical feature of M1 macrophages. At the same time, in such cells, the expression of arginase is low, an enzyme that competes with nitric oxide synthases for their substrates and, therefore, lowers NO levels [85,90]. However, in M2 macrophages, this pattern of expression in NO biology is reversed. In fact, the incubation of macrophages with IL-4 enhances arginase activity by transforming arginine into polyamines and collagen precursors, which are critical for wound healing and tissue modeling [91]. It has also been shown that the presence of apoptotic cells increases the expression of arginase II in RAW264.7 macrophages by decreasing NO production and polarizing toward an anti-inflammatory phenotype [92]. Indeed, p47 (phox-/-) mice not only decreased ROS production, but also increased arginase expression, which both indicate an important role in macrophages’ cell fate [93].

Beyond the suggested role of NOX2 and/or NO in the regulation of M2 macrophages, nuclear factor erythroid 2–related factor 2 (NRF2), a master regulator of antioxidant defense, is also associated with macrophage function [94]. As explained before, macrophages exert their activity by creating a toxic environment. Therefore, self-protective mechanisms are necessary to secure their survival, with NRF2 being the most prominent redox-sensitive transcription factor involved. A large literature body describes NRF2 regulation in the phagocytic function in various diseases of inflammatory origin. Depending on the tissue context, NRF2 deficiency can, on the one hand, lead to macrophage malfunction [95,96,97,98] or, on the other hand, its overexpression is associated with deteriorated immunological function [99,100]. In relation to M2 activation, it has been verified that a classic target of NRF2, Cu, and Zn-superoxide dismutase (SOD1) is associated with M2 polarization. In a redox-sensitive mechanism, STAT6 is activated through the oxidation of cysteine residues by SOD1-dependent H_2_O_2_ production, which leads to the M2 phenotype [101]. In this study, it was observed that the SOD1−/− mice presented an abundance of alveolar macrophages in the M1 phenotype, while the SOD1TG mice expressed mainly M2 macrophage markers. 

Finally, it should be noted that, although it seems paradoxical, the presence of ROS is also important for the activation of M2 macrophages. In an elegant study, Zhang et al. showed that the addition of an antioxidant, such as butylated hydroxyanisole (BHA), which is responsible for NOX-mediated superoxide inhibition, blocks the differentiation of M2 macrophages. This finding suggests that ROS may be implicated in early-stage M2 macrophage polarization [102].

## 3. Role of Metabolic Reprogramming and Its Redox Control in Macrophage Polarization

M1 and M2 macrophages exhibit different metabolic profiles [103]. M1 macrophages up-regulate iNOS to produce nitric oxide from arginine, and display enhanced glycolytic metabolism, pentose phosphate pathway (PPP), and fatty acid synthesis, but impaired tricarboxylic acid cycle (TCA) and mitochondrial oxidative phosphorylation (OXPHOS). In contrast, M2 macrophages metabolize arginine by arginase (Arg)-1 to produce ornithine and urea, exhibit enhanced OXPHOS and fatty acid oxidation, but impaired PPP activity [104].

Because of aerobic glycolysis activation, in M1 macrophages glucose uptake enhances and, thus, the conversion of pyruvate into lactate increases [105]. It is important to note that a break in the TCA cycle in M1 macrophages, characterized by a decreased carbon transition from citrate to alpha-ketoglutarate *(*α-KG), leads to an increase in the anaplerosis of TCA cycle intermediates through glutamine, which serves as a critical switch for M1 polarization [106]. In addition, mtROS production augments and, as a consequence of PPP activation, the NADPH formation rate, which is crucial for ROS and NO production, also increases [83,107,108]. Altogether, these metabolic events provide M1 macrophages with rapid energy and a large amount of reducing equivalents. In contrast, M2 macrophages obtain their energy in a more sustained way, particularly through fatty acid oxidation and the oxidative metabolism [105].

**Figure 3:**
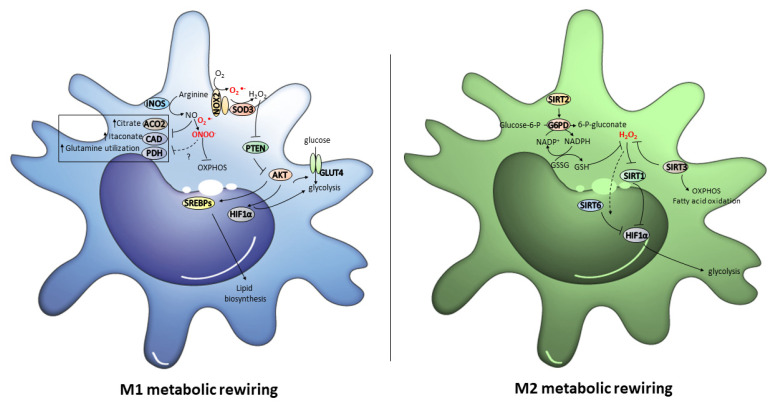
The redox signals that orchestrate M1 and M2 metabolic rewiring. In M1 macrophages, AKT activity, which might be regulated by NOX2/SOD3-derived H_2_O_2_, promotes glucose uptake by GLUT4, and activates both glycolysis and lipid biosynthesis through the up-regulation of HIF1α and SREBPs. In addition, the NO and ONOO^−^ derived from iNOS up-regulation cause metabolic rewiring by causing the inhibition of OXPHOS, ACO2, CAD and PDH. In M2 macrophages, SIRT2 and SIRT3 up-regulation promote OXPHOS, fatty acid oxidation and PPP, and H_2_O_2_ levels lower by activating antioxidant defense. Low H_2_O_2_ levels inhibit glycolysis by abrogating HIF1α through the activation of SIRT1 and the formation of the SIRT6/HIF1α complex.

Hence, the blockage of the oxidative metabolism in macrophages can drive macrophages to the M1 phenotype. Likewise, forcing the oxidative metabolism in M1 macrophages triggers the M2 phenotype [105]. However, the signaling processes that orchestrate these switches are poorly understood. Here, we assess how redox signals can contribute to macrophages polarization through the reprogramming of their metabolic status (Figure 3).

### 3.1. Redox Regulation of the Metabolic Switch to the M1 Phenotype

NOX2 is greatly activated in macrophages during acute inflammation [109]. NOX2 activation is coupled to the signaling mediated by TLR4 receptors and, thus, NOX2 deficiency down-regulates the macrophage response to LPS/IL-4 [110,111,112]. The superoxide derived from NOX2 can generate H_2_O_2_ by spontaneous dismutation, but, more often than not, this dismutation can be mediated by superoxide dismutase 3 (SOD3) in spatially confined microdomains on the cell surface. This coupling confers localized H_2_O_2_ production for redox signaling purposes [113]. Different cytosolic- or membrane-associated proteins can be targets of NOX2/SOD3-derived H_2_O_2_ on the macrophage cell surface. In particular, phosphatase and tensin homolog (PTEN), a redox-sensitive protein with multiple cysteine residues in its active site, which inhibit macrophage polarization from M1 to M2 [114,115], is particularly interesting in this regard because it is a critical inhibitor of the AKT signaling pathway [116]. 

Under oxidative stress conditions, when PTEN is inhibited AKT promotes glucose uptake by favoring glucose transporter type 4 (GLUT4) trafficking to the membrane and, thus, promoting glycolysis, one of the most relevant signatures of M1 macrophages [117]. In addition, mammalian target of rapamycin (mTOR), and particularly mTOR complex 1 (mTORC1), which is a kinase complex that consists of mTOR, the regulatory protein associated with mTOR (Raptor), and the mammalian lethal with Sec13 protein 8 (mLST8) [118], is also activated by AKT and is crucial for the activation of glycolysis, PPP, and lypid biosynthesis. This is produced via the expression of HIF1α, which induces anaerobic glycolysis by mediating the up-regulation of pyruvate dehydrogenase kinase 1 (PDK1), the inhibition of pyruvate dehydrogenase (PDH) and the pyruvate to lactate conversion, and sterol regulatory element-binding proteins (SREBPs), a family of transcription factors that regulate several critical enzymes for lipid biosynthesis [119,120,121].

On the other hand, iNOS up-regulation in M1 macrophages, which could also be orchestrated by redox signals (see Section 2.1), might be a determining factor for the redox control of its metabolic status. NO-derived from iNOS can produce high peroxynitrite levels in mitochondria, which can nitrosylate or nitrate iron sulfur proteins in the electron transport chain and inhibit electron transfer [122,123,124,125]. In fact, NO inhibits mitochondrial ETC complexes and promotes their loss in the human bone marrow-derived macrophages (BMDMs) activated with LPS + IFNγ [126]. In particular, NO regulates the abundance of the critical NADH-binding module subunits in mitochondrial Complex I [127].

Furthermore, NO also modulates metabolic remodeling in inflammatory macrophages through changes in the TCA cycle. On the one hand, NO-derived from activated macrophages cause citrate, succinate, and fumarate accumulation [126,127]. In LPS/IFNγ-stimulated BMDMs, NO reduces mitochondrial aconitase (ACO2) activity due to the disruption of its iron-sulfur cluster by, thus, causing citrate accumulation [126]. Remarkably, the [4Fe−4S]^2+^ cluster in ACO2 acts as a redox sensor to regulate its enzymatic activity because it can be oxidized and inactivated by H_2_O_2_ or, more interestingly, by NO-derived oxidative species peroxynitrite and S-nitrosoglutathione (GSNO) [128,129]. Hence, it is possible to hypothesize that NO-dependent oxidative modifications can regulate ACO2 activity during macrophages polarization. On the other hand, NO also causes itaconate accumulation, one of the metabolic hallmarks in activated macrophages [127,130]. Itaconate synthetic enzyme cis-aconitate decarboxylase (CAD) does not change in LPS/IFNγ-stimulated BMDMs. Therefore, only changes in enzyme activity can explain the dramatic accumulation of this metabolite [127]. However, whether ROS and/or RNS can play a role in regulating CAD activity remains to be explored. In any case, itaconate accumulation is a key step to initiate rewiring to the M2 phenotype because it has been well-demonstrated that itaconate can suppress the inflammatory response in M1 macrophages and, therefore, itaconate and its derivatives have become appealing therapeutic drugs against uncontrolled inflammatory processes (see Section 4). 

Together with changes in the TCA cycle, NO also inhibits pyruvate oxidation via PDH, but increases glutamine utilization in LPS/IFNγ-stimulated macrophages. This indicates a NO-dependent switch toward glutaminolysis to sustain the mitochondrial metabolism during M1 polarization [126]. The stabilization of HIF1α, which inhibits PDH and limits acetyl coenzyme A for the TCA cycle [131], can be produced by the peroxynitrite-dependent S-nitrosation of its Cys533 [132] and, in fact, compromised PDH activity has been found in the macrophages lacking Hif1α [133]. Hence, it is tentative to speculate about the role of this mechanism in M1 macrophages NO-dependent repression of PDH. However, the decreased carbon flux through PDH is HIF1α-independent in the BMDMs stimulated with LPS + IFNγ [126]. This fact suggests that the production of either N_2_O_3_ or NO_2_, derived from the reaction of NO with oxygen, could directly inactivate PDH. However, more biochemical studies are required to explain the specific role of RNS in regulating the NO-mediated glutamine rewiring in M1 macrophages.

### 3.2. Redox Regulation of the Metabolic Switch to the M2 Phenotype

NAD^+^ is as a key redox signaling molecule that catalyzes electron transfer in metabolic reduction-oxidation reactions [134]. In tissues, when nutrient availability decreases during, for instance, caloric restriction or exercise, the NAD^+^ concentration rises, but lowers in response to high levels of nutrients due to the augmented metabolic flux through glycolysis, which leads to more pyruvate formation and increases intracellular acetyl-CoA generation, the substrate for acetylation [135]. In addition, NAD^+^ concentration is also a deacetylation-rate limiting factor for NAD^+^-dependent sirtuin family activity, which exerts a key role in metabolic control [119]. SIRTs regulate the extent of acetylation and, therefore, the activity of several transcription factors or co-factors, such as peroxisome proliferator-activated receptor gamma coactivator 1-alpha (PGC-1α), forkhead Box O (FOXO) 1, FOXO3, PPARα or NF-κB, which play a central role in metabolic regulation [136].

In the presence of active SIRT1, inflammation is inhibited because NF-κB-subunit p65 requires acetylation by p300 to transcribe inflammatory genes, such as TNF-α [137]. Moreover, the deacetylase activity of SIRT1 up-regulates PGC-1α to favor fatty acid oxidation, but destabilizes HIF-1α by inhibiting glycolytic metabolism, which are two critical features of M2 macrophages [138,139,140]. Accordingly, SIRT1 activation down-regulates LPS-mediated NF-κB activity by inhibiting p65 acetylation and the expression of M1 genes, and abrogates the HIF1α-dependent transcriptional program, which is responsible for heightened glycolysis in macrophages [141,142,143]. In fact, SIRT1 expression increases after caloric restriction in visceral fat by augmenting the levels of M2 polarized macrophages [144]. It is noteworthy that high H_2_O_2_ levels induce the proteasomal degradation of SIRT1, desumoylation, and enzyme inactivation which, thus, indicate the ability of oxidative species to modulate SIRT1 activity and cell metabolism [145]. Interestingly, oxidative stress may cause a dysregulation in normal SIRT1 functioning. It has been observed that in response to oxidative stress, SIRT1 is redistributed at the chromatin level, causing transcriptional changes [146]. Indeed, human monocytes exposed to a high H_2_O_2_ dose have led to a significant reduction in SIRT1 activity and reduced SIRT1 gene and protein expressions [147]. This scenario suggests that lowering H_2_O_2_ levels in macrophages could contribute to M2 polarization. 

SIRT2, SIRT3, and SIRT6 also strongly impact the metabolic regulation and reprogramming of macrophages. SIRT2 inhibits glycolysis and promotes lipolysis, gluconeogenesis and PPP in cell- and disease-dependent manners [148]. SIRT3 is the main mitochondrial deacetylase and a major regulator of cell metabolism. SIRT3 promotes TCA and electron transport chain, ketogenesis, and fatty acid oxidation, but inhibits oxidative stress by activating isocitrate dehydrogenase 2 (IDH2) and SOD2 [149,150,151,152]. SIRT6 is located in the cell nucleus and has been shown to function by regulating the chromatin structure, as well as the recruitment and activity of key transcriptional factors, such as NF-κB, NRF2, PGC-1α, or HIF-1α [153,154,155]. 

SIRT2 deficiency in mice causes increased susceptibility to experimental colitis by increasing NF-κB acetylation and reducing M2-associated anti-inflammatory pathways [156]. SIRT3-deficient mice develop severer acute lung injury compared to wild-type mice. The macrophages obtained from these mice exhibit significant alterations to mitochondrial bioenergetic and redox homeostasis, which have been associated with a proinflammatory phenotype [157]. Furthermore, SIRT3-overexpressing Mϕs transfusion induces M2 polarization, and alleviates inflammation, apoptosis, and crystal deposition in glyoxylate-induced kidney stones in mice [158]. Strikingly, SIRT2/3^−/−^ macrophages favor fatty acid oxidation over glycolysis, and SIRT2/3^−/−^ mice are robustly protected from endotoxemia [148]. SIRT6 drives macrophage polarization toward M2 by promoting IL-4 production, and its deficiency elicits macrophage polarization toward an M1 phenotype and delays wound healing in mice [159,160,161].

Remarkably, under oxidative stress conditions, SIRT2 has been shown to deacetylate and activate G6PD, a key enzyme in PPP that produces NADPH to maintain glutathione (GSH) in its reduced form [153]. In addition, oxidative stress conditions lead to the deacetylation and activation of PGAM2 by SIRT2, which allows the cell to more easily respond to stress [162]. Furthermore, direct oxidation of two redox sensitive cysteine sites, Cys221 and Cys224, significantly reduced SIRT2 enzymatic activity leading to NF-κB acetylation during exaggerated hyper-inflammation of obesity with sepsis [163].

On the other hand, increased ROS levels not only stimulate SIRT3 transcription to inhibit ROS-mediated HIF-1α stabilization [153], but also cause S-sulfenylation of SIRT6, a corepressor of HIF-1α. In particular, of the five cysteine residues in SIRT6, Cys18 is exclusively and highly *S*-sulfenylated in cells exposed to H_2_O_2_, which causes SIRT6 to interact with HIF-1α and show a very interesting redox-dependent mechanism to regulate HIF-1α transcriptional activity through the reversible formation of the disulfide-linked SIRT6–HIF-1α complex [155]. In addition, direct and reversible cysteine thiol 144 oxidation in SIRT6 during sepsis hyperinflammation regulates its glycolytic function, contributing to immunometabolic paralysis in human and mouse sepsis monocytes [164].

Taken together, these findings suggest that the ROS-dependent modulation of SIRT2, SIRT3, and SIRT6 activity could play a key role in the transition phase of the M1 to M2 phenotype to orchestrate harmonious rewiring between the two metabolic faces of macrophages.

## 4. Modulating Macrophage Function by Redox Signals: A Therapeutic Point of View

This review provides details of macrophage polarization being a highly regulated process through different signaling pathways, in which redox signaling plays a fundamental role. In this context, and knowing that the interruption of redox signaling and oxidative stress are described in the onset and progression of many diseases, macrophages may represent an appealing therapeutic target in diseases with an inflammatory component. In fact, therapies that aim to regulate the inflammatory state of macrophages specifically through the redox state are increasingly seen as powerful tools to counteract the deleterious effects of their activation. Here, we review novel therapeutic approaches based on redox mechanisms in association with macrophage plasticity, which could be exploited in the context of acute inflammation. 

Among the different therapeutic strategies, the NRF2 activity regulation has been the target used by most authors. Resveratrol, a potent NRF2 activator [165], suppresses macrophage activation in LPS-induced AKI. In AKI, macrophages acquire an M1 phenotype (see Section 1.2.3), but resveratrol administration causes significantly augmented IL-10 levels in association with the anti-inflammatory effects mediated by M2 macrophages [166]. Interestingly, the dual AMP-activated protein kinase (AMPK) and NRF2 activation by drug HP-1c, a hybrid of telmisartan and 2-(1-Hydroxypentyl)-benzoate (HPBA), exerts neuroprotective effects by promoting the M2 microglial phenotype and reducing oxidative stress in ischemic stroke in rats [167]. It is important to note that other molecules, such as quercetin, quercitrin, and dehydroepiandrosterone (DHEA), are able to suppress inflammatory responses through NRF2 activation in LPS-stimulated RAW 264.7 macrophages [168,169]. Yet, whether the antioxidant properties of these molecules modulate macrophage polarization is a question that remains to be explored.

Apart from using general NRF2 activation as a potential therapy, other studies have been conducted based on specific targets of this transcription factor in different inflammatory processes. Glutaredoxin (GRX) 1, a thiol-disulfide oxidoreductase that specifically catalyzes the reduction of S-glutathionylated substrates, is known to be a regulator of inflammatory disease models [170,171]. Remarkably, Guo et al. have demonstrated that macrophage-specific Grx1 deficiency alleviates acute lung injury inflammation [172]. In their work, ROS induce S-glutathionylation of fatty acid binding protein (FABP5) in macrophages, which increases nuclear FABP5 levels, activates PPARβ/δ, and abrogates inflammation in the macrophages incubated with LPS [172]. Therefore, if we bear in mind that the GRX1 levels in the M1-type macrophages were high [173], the use of different specific inhibitors of GRX1, such as CWR-J02 or APR-246/MQ, to modulate the macrophage polarization process during inflammation would appear to be a promising therapeutic strategy [171,174]. Strikingly, the administration of GRX2, an isoform that, unlike GRX1, is not inhibited by the oxidation of structural Cys residues [175], lowers NO levels in macrophages and alleviates asthma-like acute airway inflammation in mice [176]. So it would seem that the reversible oxidation and glutathionylation of the proteins catalyzed by different GRX isoforms play a complex role by regulating macrophage function, which should be fully clarified before approaching a therapy based on this system.

Interestingly, NRF2 activation has also been associated with the modulation of macrophage phenotype by targeting the metabolic signature of these cells. In this context, the immunomodulatory and antioxidant properties of itaconate have been highlighted. Itaconate is a mitochondrial metabolite formed by the catalytic activity of aconitate decarboxylase 1 (ACOD1), which uses cis-aconitate from the TCA cycle to produce itaconate [177]. This molecule is able to mediate the transformation of the pro- to the anti-inflammatory phenotype in macrophages via different mechanisms, including NRF2 activation by the alkylation of kelch-like ECH-associated protein 1 (KEAP1), the inhibition of aerobic glycolysis by targeting glyceraldehyde-3-phosphate dehydrogenase and fructose-bisphosphate aldolase A, the inhibition of succinate dehydrogenase and the blockade of inhibitor of nuclear factor-κB *ζ* (I*κ*B*ζ*) translation [178]. Itaconate binds and exerts strong inhibitory effects on key inflammatory signaling regulators by covalently modifying cysteine residues due to its electrophilic properties [179,180]. However, the high polarity and low electrophilicity of unmodified itaconate confer it weak cell permeability to fulfill therapeutic purposes. Therefore, itaconate-based therapeutic efforts have focused on developing derivatives, which could be potent and safe pharmacological agents to treat inflammatory disorders [181]. Indeed, itaconate derivative dimethyl itaconate (DI) exhibits high membrane-permeable ability and, importantly, marked acceptor reactivity, which causes potent NRF2 activation and the significant depletion of glutathione levels [182]. However, the rapid degradation or further metabolization of DI impairs being converted into intracellular itaconate [183]. In contrast, a new itaconate derivative, 4-octyl itaconate (4-OI), is resistant to esterase hydrolysis. However, its electrophilic properties are lower [182]. 

Many studies have reported the beneficial effects of itaconate or its derivative in the acute inflammation context. DI administration enhances survival rate, decreases TNF-α and IL-6, and ameliorates lung injury in septic mice and BMDMs by promoting the expression of NRF2 and its downstream targets heme oxygenase (HO-1) and NAD(P)H dehydrogenase quinone 1 (NQO-1) [184,185]. Similar results have been obtained with 4-OI, which has a dramatic effect by inhibiting the expressions of IL-β, IL-6, and TNF-α, and counteracting ROS production in acute lung injury and acute respiratory distress syndrome [186]. Furthermore, after influenza A virus infection in mice, DI and 4OI treatments lower ROS levels and STAT1 phosphorylation, and reduce pulmonary inflammation and mortality [187]. It is noteworthy that pretreatment with DI suppresses iNOS protein expression, IL-6 secretion and, importantly, succinate dehydrogenase (SDH) activity, which induce the accumulation of succinate in LPS + IFN-γ activated macrophages [130]. SDH can oxidize succinate and produce reduced coenzyme Q, which passes electrons to complex I and generates mtROS [178]. Thus, the inhibitory effect on SDH not only impairs succinate processing, but also blunts mtROS production [130]. Hence, the protective effects of itaconate-based drugs on macrophages are associated with redox-based mechanisms, which cause functional changes in these cells that lead to inflammatory and metabolic rewiring.

Finally, other signaling pathways other than NRF2 have also been explored as potential therapeutic targets. Nattokinase (NK) is a serine protease that causes LPS-induced suppression of TLR4 and NOX2 activation in RAW264.7 macrophages and, thus, represses ROS production, MAPK activation and NF-κB translocation from the cytoplasm to the nucleus [188]. In a very similar mechanism, ACY-1215, a histone deacetylase 6 (HDAC6) selective inhibitor, inhibits ROS generation in LPS-stimulated RAW264.7 cells by suppressing the activation of the TLR4-MAPK/NF-κB signaling pathway [189]. Additionally, rhein administration attenuates inflammatory responses by mediating the polarization of macrophages from the M1 to the M2 phenotype in a redox mechanism by the inactivation of nucleotide-binding oligomerization domain, leucine rich repeat and pyrin domain containing Protein 3 (NLRP3) inflammasome, the inhibition of NOX2 subunits expression and translocation, and the up-regulation of the NRF2-HO1-NQO1 pathway [190]. Recently, it has also been observed that treatment with nano-designed carbon monoxide donor SMA/CORM2 triggers macrophage reprogramming toward M2 phenotype via HIF-1α suppression, conferring protection against APAP induced liver injury [191]. 

## 5. Conclusions

Macrophages are highly plastic cells that perform essential functions in the inflammatory process. During inflammation, macrophage polarization and reprogramming dynamically occur to ensure an adequate balance between different phenotypes depending on the inflammatory phase. This fine-tuned equilibrium is disrupted in pathological situations. 

The coordinate action of various inflammatory signals is critical for regulating macrophage polarization, and it is in this context where redox signaling plays a key role. Although a continuum of heterogeneous functional macrophage phenotypes appears during the course of inflammatory disorders, M1 and M2 phenotypes are considered the archetypal ends of this spectrum. M1 and M2 macrophages are characterized by a specific gene transcription and metabolic profiles, which are critically maintained by redox signals. Indeed changes in the redox state of macrophages decisively contribute to maintain a particular molecular signature in these cells by activating o repressing redox-sensitive inflammatory and metabolic pathways.

M1 macrophages up-regulate NOX2, iNOS, SYNCRIP, and TRAF6, which increase ROS and RNS production that, in turn, sustains the inflammatory response, phagocytosis and cytotoxicity activity against pathogens. Moreover, increased ROS and RNS levels activate glycolysis and lipid biosynthesis, but inhibit TCA and OXPHOS, a key feature of M1 macrophages. In contrast, M2 macrophages down-regulate NOX2, iNOS, and SYNCRIP and/or up-regulate arginase and SOD1 to counteract ROS and RNS, and to favor the anti-inflammatory and tissue repair response. Low ROS and RNS levels favor the activation of a metabolism profile that obtains energy in a more sustained way, particularly through oxidative metabolism and fatty acid oxidation.

Many of these redox mechanisms represent appealing therapeutic targets in diseases with an inflammatory component. In particular, the regulation of Nrf2 activity or its transcriptional targets can be especially promising because this protein seems to be located at the crossroads between metabolic and inflammatory regulation.

To conclude, both inflammatory and metabolic rewiring are two interrelated processes that interact to adapt the biology of macrophages to the surrounding microenvironment. Redox signaling acts as a master regulator of this complex interaction and is not yet fully understood. However, studying this interplay from a redox perspective guarantees the discovery of new therapeutic targets to treat acute inflammatory processes.
